# Data processing and analysis with the *autoPROC* toolbox

**DOI:** 10.1107/S0907444911007773

**Published:** 2011-03-18

**Authors:** Clemens Vonrhein, Claus Flensburg, Peter Keller, Andrew Sharff, Oliver Smart, Wlodek Paciorek, Thomas Womack, Gérard Bricogne

**Affiliations:** a Global Phasing Ltd, Sheraton House, Castle Park, Cambridge CB3 0AX, England

**Keywords:** *autoPROC*, data processing

## Abstract

A typical diffraction experiment will generate many images and data sets from different crystals in a very short time. This creates a challenge for the high-throughput operation of modern synchrotron beamlines as well as for the subsequent data processing. Novice users in particular may feel overwhelmed by the tables, plots and numbers that the different data-processing programs and software packages present to them. Here, some of the more common problems that a user has to deal with when processing a set of images that will finally make up a processed data set are shown, concentrating on difficulties that may often show up during the first steps along the path of turning the experiment (*i.e.* data collection) into a model (*i.e.* interpreted electron density). Difficulties such as unexpected crystal forms, issues in crystal handling and suboptimal choices of data-collection strategies can often be dealt with, or at least diagnosed, by analysing specific data characteristics during processing. In the end, one wants to distinguish problems over which one has no immediate control once the experiment is finished from problems that can be remedied *a posteriori*. A new software package, *autoPROC*, is also presented that combines third-party processing programs with new tools and an automated workflow script that is intended to provide users with both guidance and insight into the offline processing of data affected by the difficulties mentioned above, with particular emphasis on the automated treatment of multi-sweep data sets collected on multi-axis goniostats.

## Abstract 2

Typical topics and problems encountered during data processing of diffraction experiments are discussed and the tools provided in the *autoPROC* software are described.

## 1. Introduction

In an ideal world, a typical diffraction experiment would produce a set of images showing (i) nice diffraction, (ii) well separated lunes, (iii) perfect spot shapes, (iv) only one crystal lattice, (v) multiple measurements at every (*hkl*) within the diffracting range of the crystal and (vi) well behaved statistics. In the real world, we often have to deal with diffraction experiments that fail in some or nearly all of the above categories. This may result in the structure not being solvable at all (by either molecular replacement or experimental phasing) or in important parts of the electron density remaining uninterpretable. At this point, it is often beneficial to look at some common problems that might have been overlooked during the initial data-processing steps.

## 2. Beam centre

Errors in defining the beam centre according to the coordinate systems used by the integration program probably constitute the most frequent cause of failure in indexing a given set of images. Although the values given in the image header are usually correct, the coordinate convention used is often not uniquely specified (Sauter *et al.*, 2004 ▶; a notable exception is the imgCIF/CBF format; Bernstein & Hammersley, 2005 ▶). Furthermore, nonstandard items, as well as wrong values, are not uncommon in image headers, making it necessary to provide the correct values through a separate program-specific site-definition file or processing template.

All this can be confusing, especially for a novice user wanting to process data collected on an unfamiliar beamline. Considering the importance of a correct beam centre for indexing, one needs to establish (i) whether the beam-centre coordinates in the header are correct and (ii) the convention according to which they are given.

In normal circumstances, detector manufacturers and beamline scientists will have done their best to ensure that this information is accurate and easily accessible in the form required by the various processing packages, but this information may not have been recorded in a timely manner by the user. Other difficulties may arise from the fact that for certain instrumental configurations the beam centre may drift as a function of ambient temperature. Changes to beamline software may also on occasion break a previously established convention for the storage of beam-centre coordinates. It is often possible to determine the correct axis convention by analysing the concentricity of some image features around the purported beam centre under all eight conventions; more details and some results are discussed in §[Sec sec8.2]8.2.

## 3. Multiple lattices

**Figure 1:**
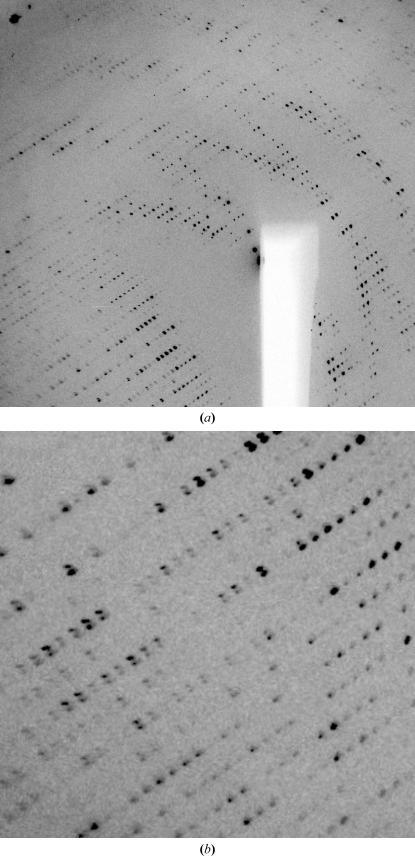
Example of multiple lattices: orientation (*a*) shows distinct lunes for two lattices of nearly equal strength, whereas in orientation (*b*) reflections from the two lattices are nearly completely overlapping.

Crystals often show multiple lattices during data collection, as seen in Fig. 1 ▶. These could be a consequence of split crystals, of a satellite crystal being present in the loop or of a specific relation between domains, *i.e.* nonmerohedral twinning (for a recent review on twinning, see Parsons, 2003 ▶).

Especially when there is a distinct relation between the two (or more lattices), the relative strength of the different lattices and the extent of overlap are not always visible on the first image. It is therefore important to inspect several images taken at different positions of the rotation axis (*e.g.* at 45° and 90°Å from the starting position). Sometimes, a second lattice might only become apparent when a new part of the crystal (or loop/mount) moves into the beam during rotation of the crystal. The degree to which such additional lattices can cause problems later during indexing or integration can sometimes be reduced by recentring the crystal so that the beam hits a different part of it. Recent developments at synchrotrons to implement so-called grid, line or mesh scans will help in finding the best-ordered part of a crystal (Bowler *et al.*, 2010 ▶).

## 4. Consistent indexing

Diffraction data sets frequently comprise several ‘sweeps’ (*i.e.* sets of images with contiguous rotation ranges around a common axis) that would be processed, and in particular indexed, independently in separate invocations of programs such as *MOSFLM* and *XDS*. It is often important to ensure that these various sweeps be indexed in a consistent manner. The first level of consistency is that all sweeps be indexed equivalently, *i.e.* up to the point-group symmetry of the crystal. Such a need is obvious in the case of multi-wavelength data sets, where failing to fulfil this consistency criterion when indexing the sweeps corresponding to the various wavelengths would create chaos in the subsequent use of the data for MAD phasing. The same applies to the merging of low- and high-intensity passes, as well as to that of multi-sweep data sets collected for various crystal orientations with a multi-axis goniostat.

A more demanding level of consistency, which is exact identity rather than equivalence up to a point-group operation, is required if it is desired to make use of the empirical absorption correction in *SCALA* by means of a common absorption surface defined in the crystal frame. This can play a crucial role in, for example, sulfur-SAD phasing at long wavelengths, where anomalous differences are weak and absorption is strong. In this case, the known relations between the crystal orientations attached to the various sweeps must be taken into account in the enforcement of consistent indexing, and this requires a complete specification of the instrument, in particular of the goniostat. This is discussed further in §[Sec sec8.4]8.4.

## 5. Ice rings

The presence of ice rings can have a strong impact on the success of indexing, integration, scaling, structure solution and refinement. If they cannot be avoided during cryocooling of the crystal, the affected resolution ranges should be excluded from all processing steps. An alternate approach involving preprocessing of the raw diffraction images has recently been proposed by Chapman & Somasundaram (2010 ▶).

**Figure 2:**
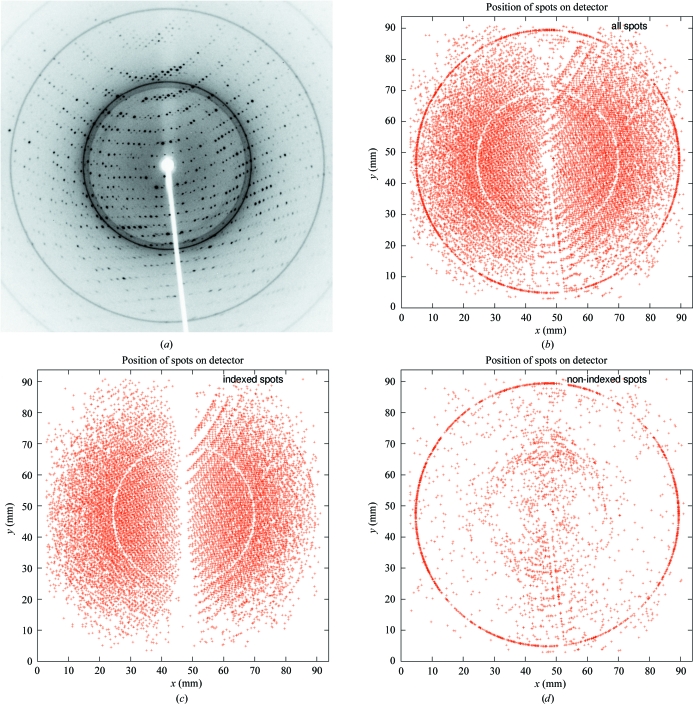
Classification of spots for ice rings: (*a*) original single image; (*b*) spots represented by a red cross as collected from a series of images; (*c*) selection of spots that could be used for indexing (the ‘white’ circle corresponds to a strong ice ring preventing any spots being found); (*d*) remaining unindexed spots.

During indexing, all found spots can be classified as either belonging to the found indexing solution or left over. This allows an easy visual check for the presence of ice rings (see Fig. 2 ▶).

## 6. Looking at the reflection file

The last step should be to look at the reflection file (or files) using the *CCP*4 program *HKLVIEW* for displaying pseudo-precession pictures of a single column of an MTZ file con­taining these reflections. Tools within the *HKLVIEW* interface allow zooming and scaling of the reflection spots (which are represented by square boxes in greyscale, with their size and colour related to the intensity value of the reflection). A tool has recently been added to the *PHENIX* package to create similar pictures using raw diffraction images (Sauter, 2011 ▶).

**Figure 3:**
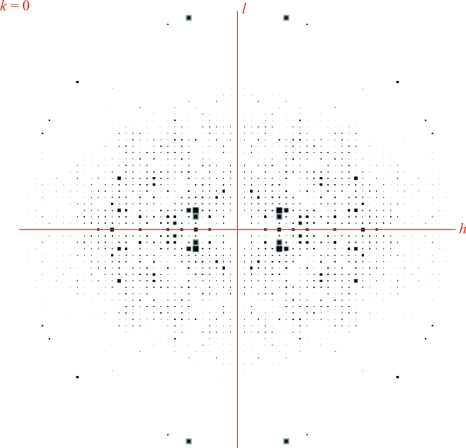
Pseudo-precession photograph using *HKLVIEW*.

Several basic features of reflections can be seen in these views. Fig. 3 ▶ shows some unexpectedly strong reflections at high resolution. These coincide with typical ice-ring resolutions at around 2.15 Å and are symptomatic of problems during data processing (incomplete exclusion of ice rings) or merging (failure of outlier detection).

### 6.1. Anisotropic diffraction limits

The falloff in intensity along different directions in the *h*00, 0*k*0 or 00*l* planes highlights a common situation: the volume of crystal in the beam can change when rotating the crystal during the experiment and the order within the crystal may be better in some directions than in others. This anisotropy causes a systematic loss of accuracy for a subset of the data and can lead to subsequent problems when using methods that assume a more isotropic behaviour of the diffraction data, such as molecular replacement, substructure detection using normalized structure factors and real-space methods such as density modification.

Correcting for anisotropy can be performed with *SHARP* (Bricogne *et al.*, 2003 ▶), *SFCHECK* (Vaguine *et al.*, 1999 ▶), *Phaser* (McCoy *et al.*, 2007 ▶) or the *Diffraction Anisotropy Server* (Strong *et al.*, 2006 ▶).

### 6.2. Problematic resolution shells

There should be a smooth falloff in intensity values with resolution: lower resolution reflections are typically stronger than high-resolution reflections (see the Wilson plot; Wilson, 1949 ▶). Any deviation from this is highly suspicious: exceptionally strong reflections reported at higher resolution could arise from the integration step having mishandled the high background associated with diffuse ice diffraction. Analysing the resolution of these strong high-resolution reflections can point towards such an ice-ring problem (see example in Fig. 3 ▶). Typical ice-ring resolutions include 3.90, 3.67, 3.44, 2.67, 2.25, 2.07, 1.95, 1.92, 1.88 and 1.72 Å (Garman & Schneider, 1997 ▶; Chapman & Somasundaram, 2010 ▶).

### 6.3. Detector overloads and missing low-resolution data

Detector overloads will result in the corresponding measurements being rejected during data processing. The consequences of overloads are not random: they affect the strongest reflections, which tend to occur at low resolution. While these reflections might be few in number, they are vital for the successful use of a number of important methods such as molecular replacement, solvent flattening in density modification or bulk-solvent modelling in refinement (Evans *et al.*, 2000 ▶). When overloads are a problem, a separate low-intensity sweep should be recorded and merged into the data set during processing. Using an attenuated beam rather than a shorter exposure time, together with a larger angular range per image, would be likely to yield better measurements of low-resolution reflections by better mitigating instrumental errors associated with noncontinuous (start/stop) crystal rotation.

## 7. Expect the unexpected

Even a set of very good images, resulting in a high-quality data set with very good statistics, might not enable the solution of the structure. This is especially frustrating if, for example, the anomalous signal seems to be of good quality to high resolution or a highly homologous structure exists in the PDB. It is possible that the purified protein is an expression artefact rather than the protein that was intended to be isolated. It can be useful to check the PDB (Berman *et al.*, 2006 ▶) for entries with a similar unit cell and space group. If such an entry exists (especially of a protein that is very similar or identical to one that is natively produced by the expression system used to prepare the sample), molecular replacement can be used to confirm or eliminate it as a possible solution. Several cases are known to us in which data were collected from crystals of in­organic pyrophosphatase from *Escherichia coli* (Kankare *et al.*, 1996 ▶) in the belief that they consisted of something else. Other examples are given by Lohkamp & Dobritzsch (2008 ▶) and Veesler *et al.* (2008 ▶).

## 8. The *autoPROC* software

**Figure 4:**
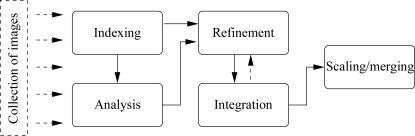
Typical offline data-processing steps.

To help users through the various steps from images to a fully processed, scaled and merged data set, various comprehensive software packages have been developed (Pflugrath, 1999 ▶; Holton & Alber, 2004 ▶; Sauter *et al.*, 2004 ▶; Minor *et al.*, 2006 ▶; Winter, 2010 ▶). Over the last five years, we have developed a set of programs that make up the *autoPROC* framework together with several third-party programs. The collection of modules that make up this framework are intended as an offline tool for the fully automatic processing of diffraction images from single-sweep or multi-sweep experiments (*e.g.* multi-wavelength MAD, low-resolution and high-resolution passes, inverse-beam or interleaved-wavelength data collection). The typical steps during this process involve (i) image analysis; (ii) spot search; (iii) indexing; (iv) initial analysis of diffraction quality and detector parameters; (v) refinement of initial unit-cell parameters, orientation and mosaicity; (vi) determination of the most likely space group; (vii) integration of all images and (viii) scaling and merging of integrated intensities (see Fig. 4 ▶).

Since June 2005, *autoPROC* has been released to members of the Global Phasing industrial consortium as well as various academic beta testers and synchrotron beamlines. It has been extensively used and incorporated in high-throughput pipelines and has seen several updates since then. The latest version is expected to be released to academic users in the first quarter of 2011.

### 8.1. Implementation

*autoPROC* is implemented as a series of modules for the various steps shown in Fig. 4 ▶. Each module is clearly separated from the others, with a defined set of input and output parameters. The original implementation used mainly *MOSFLM* (Leslie, 1992 ▶) and *SCALA* (Evans, 1997 ▶) as the pipeline components. Subsequent developments added support for *XDS* (Kabsch, 2010 ▶) as the data-processing engine and *POINTLESS* (Evans, 2006 ▶) for space-group determination. Several programs from the *CCP*4 suite (Collaborative Com­putational Project, Number 4 , 1994 ▶) are also used within the pipeline. Additional software components developed exclusively for *autoPROC* are available to add further functionality and robustness. A collection of auxiliary tools is provided to help the user during automated data processing. Execution of programs is mainly command-driven and in its simplest form can take place through a single command (using all default settings)

% process

Several mechanisms are provided to fine-tune the data processing and decision-making for a particular data set, a specific beamline or instrument, a series of data collections coming from a known crystal form or challenging projects that might require nonstandard parameters. Owing to the many data sets that a typical synchrotron trip can yield, a macro facility is implemented to group a collection of settings to enable easy and fast application of *autoPROC* to a large collection of data sets. This also allows easy incorporation of the software into a larger in-house pipeline, *e.g.* in drug-discovery programs or structure-based drug design.

### 8.2. Determining the beam centre

The *GETBEAM* program is provided in order to help the user to understand the relationship between the image-header values for a specific instrument or beamline and the values expected by the integration program (as driven through *autoPROC*). It allows the testing of coordinate conventions, the analysis of direct-beam shots and the refinement of input beam-centre coordinate values.

**Figure 5:**
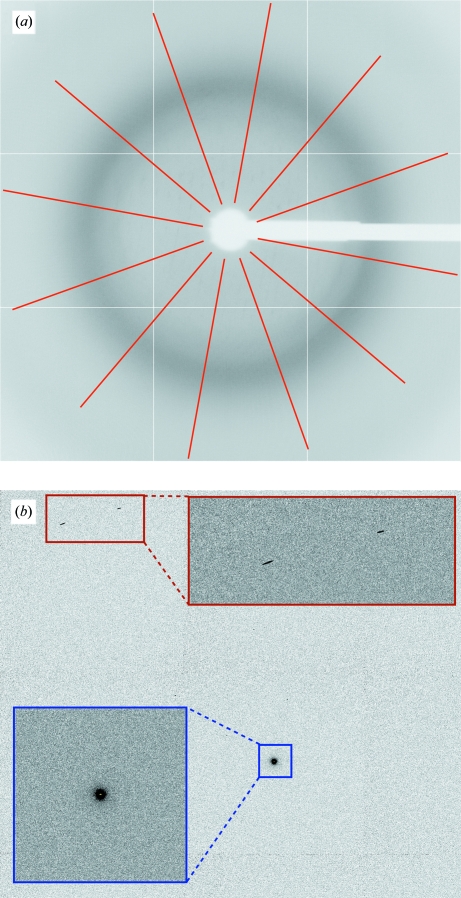
Using *GETBEAM* to help define direct-beam coordinates: (*a*) background-only image for 1vq0 (JCSG, 2006 ▶) with lines used for calculating correlations between opposite areas; (*b*) part of a direct-beam shot image with enlarged areas around the direct-beam position (blue) and some rogue high-value pixels (red).

If a direct-beam shot image is given, the largest pixel value in the image array is used. The search algorithm is restrained to the initial beam-centre value (which is usually obtained from the image header) in order to avoid finding a rogue pixel or zinger, as shown in Fig. 5 ▶.

When no direct-beam shot image is available, a series of normal images can be used. To remove the effect of diffraction spots on these images, a so-called underlay image is constructed (Pflugrath, 1999 ▶): for this, the smallest pixel value found in all images at each position is taken. The final image should be void of actual diffraction spots if several images wide enough apart in oscillation angle are used. Ideally, the only remaining feature of this image should be the diffuse background coming mainly from the solvent in and around the crystal. In a setting where the direct beam is perpendicular to the detector surface this should be a radially symmetric distribution with the direct-beam coordinates at its centre. Fig. 5 ▶ shows a series of lines emanating from the current beam centre constructed in order to calculate the correlation of pixel values between opposite lines. This score is used in either deciding which of the eight possible choices of origin is the most likely or, if well defined features with circular symmetry such as ice rings are present, to refine an initial beam-centre position.

A collection of 356 data sets (JCSG, 2006 ▶) collected between October 2001 and September 2010 was used to analyse the usefulness of this method to determine the most likely coordinate convention that the beam-centre values recorded in an image header refer to. Nearly half of these data sets (170) had the beam centre recorded as the midpoint of the image and were excluded from further analysis. Of the remaining 186 data sets, three could not be indexed correctly. For the remaining 183 data sets the average distance between refined beam-centre values and the values recorded in the header was 67.9 pixels. On the other hand, the same average distance after using *GETBEAM* was only 5.4 pixels. This clearly shows the benefit of testing for the coordinate con­vention of header values using this approach.

### 8.3. Multiple lattices

**Figure 6:**
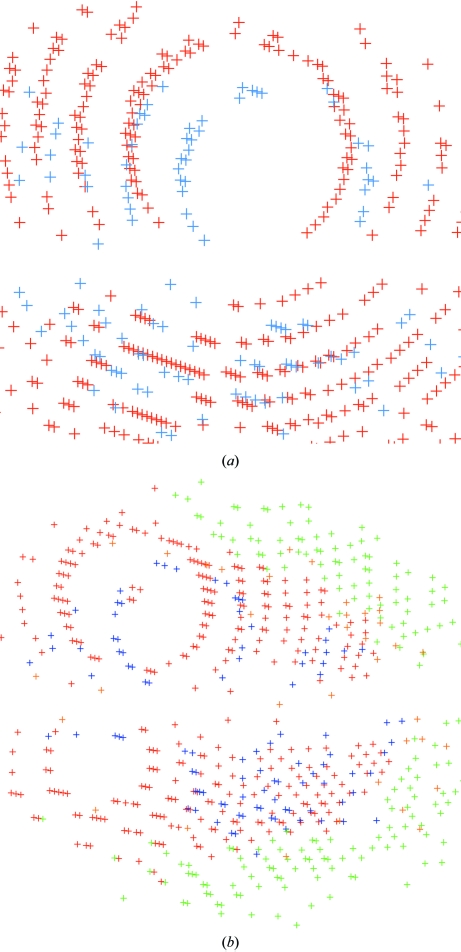
Visualization of multiple lattices in 1vk2 (JCSG, 2006 ▶) by *autoPROC*: both pictures show ‘lattices’ in different colours. The two main lattices are shown in red and blue.

*autoPROC* allows the detection of multiple lattices and robust indexing of the main lattice (see Fig. 6 ▶). This is achieved through an iterative selection of spots matching the current indexing matrix. This approach is similar to that presented by Sauter & Poon (2010 ▶). Spots that clearly do not match the current orientation matrix are pooled for a second round of indexing: in this way, additional lattices can be detected automatically and their relation to the main lattice can be analysed. Furthermore, spots that cannot be indexed at all within any of the orientation matrices obtained are used to search for possible ice rings in the diffraction images (Fig. 2 ▶).

**Figure 7:**
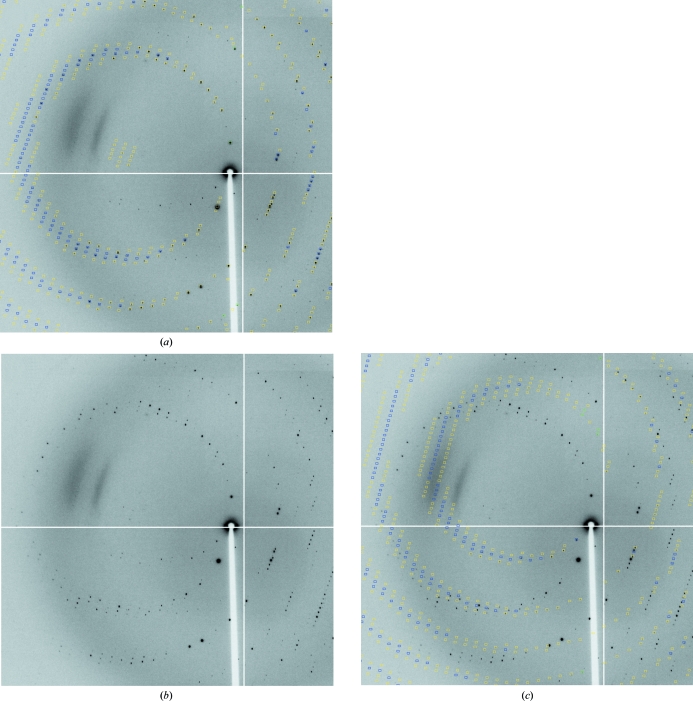
Determining separate orientation matrices for different lattices in 1vk2 (JCSG, 2006 ▶): (*a*) predictions for the main lattice (fulls, blue; partials, yellow; too wide in ϕ, green); (*b*) diffraction image without predictions; (*c*) minor lattice predictions.

Data processing is performed using the best orientation obtained for the highest populated lattice (see Fig. 7 ▶), but the user could also select any of the minor lattices for integration. However, with the current integration programs implemented in *autoPROC* there still remains the possibility of wrongly integrating spots that overlap between the lattices or of the parameter refinement switching between lattices for specific crystal orientations (where the lattices are not separated on the data images). Further developments will aim to address the problem of integrating and processing overlapped spots in the presence of multiple lattices.

### 8.4. Consistent indexing

**Figure 8:**
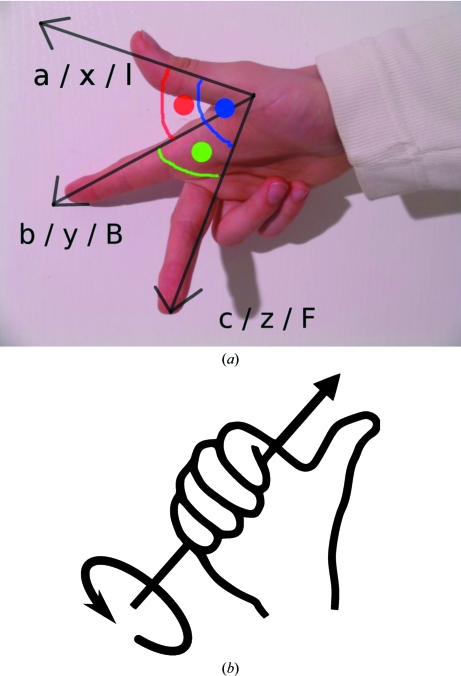
Visualization of a right-handed coordinate system and of the right-hand rule for rotation around an axis (from http://en.wikipedia.org/wiki/Right-hand_rule).

In all cases where exact consistency of indexing is required between the individual sweeps of a multi-sweep data set in which the action of a goniostat has been involved, *autoPROC* uses an auxiliary program *KAPPAROT* to calculate the motions of general goniostats (Kappa and Eulerian) as well as those of 2θ arms if applicable. Instrument definitions are flexible and follow simple rules regarding right-handed co­ordinate systems and axis rotations (Fig. 8 ▶).

**Figure 9:**
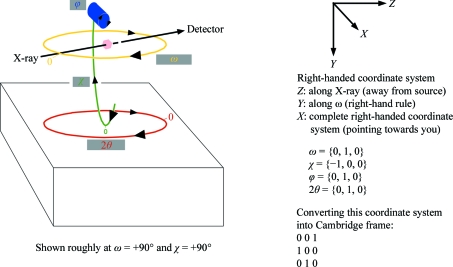
Defining goniostat axes. The so-called Cambridge reference frame follows the definition of *MOSFLM* (Leslie, 1992 ▶).

Based on this description (see the example in Fig. 9 ▶), the well defined relation between separate sweeps is maintained and the resulting orientation matrices will be correctly related through the known goniostat motions, provided the complete set of required goniostat angles is written into each image header.

This is achieved by using a general treatment of multi-axis goniometry and detector geometry first proposed by Thomas (1986 ▶, 1990 ▶, 1992 ▶) and used in the EEC Workshop on Position-Sensitive Detector Software (Bricogne, 1986 ▶, 1987 ▶) to convert the initial version of the *MADNES* program, originally written for the Nonius FAST detector (Messerschmidt & Pflugrath, 1987 ▶), into an instrument-independent package (Pflugrath, 1997 ▶). The same treatment was subsequently implemented in d*TREK (Pflugrath, 1999 ▶) and extended by Paciorek *et al.* (1999 ▶).

### 8.5. Visualization

**Figure 10:**
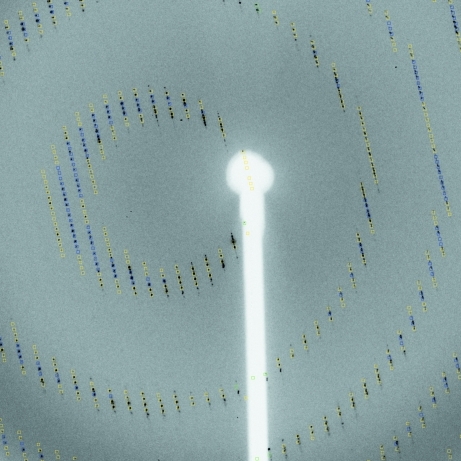
Visualizing *XDS* results with *MOSFLM*: the orientation matrix from *XDS* is transformed by *autoPROC* into *MOSFLM* format, together with distance, beam centre and mosaicity. The resulting descriptions can directly be loaded into *MOSFLM*, where interactive tools are then available for showing predictions, analysing the beam centre or ice rings, adjusting mosaicity values *etc*. (3lov; JCSG, 2006 ▶; blue, fulls; yellow, partials; green, too wide in ϕ).

To check the results obtained during data processing, *autoPROC* converts the *XDS* orientation information into a form suitable for use with *MOSFLM* (as seen in Fig. 10 ▶). This allows visual inspection of the predictions made on the basis of the current orientation matrix, unit-cell parameters, mosaicity *etc*.

### 8.6. Results

**Figure 11:**
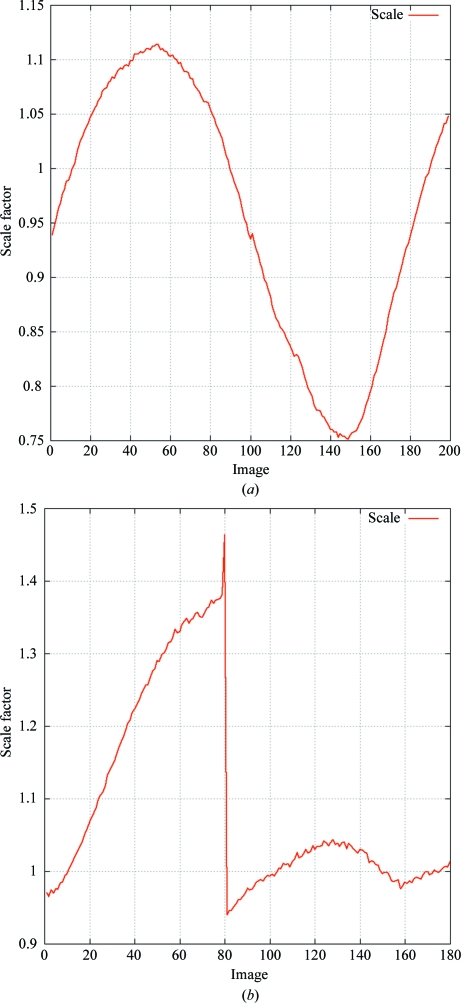
Scale factor based on background scatter *versus* image number from *XDS*. These plots are generated automatically by *autoPROC*. (*a*) shows a typical example of the different scattering power of a crystal during a full 180° rotation; (*b*) shows an event between two images of 2eth (JCSG, 2006 ▶).

To keep the amount of information given to the user at a minimum, the most important results (indexing solution, space-group determination, merging statistics, automatic determination of high-resolution limit), together with some notes and warning messages, are reported. Several statistics as well as refined parameters are given either as a function of resolution or as a function of image number. The former allow decisions to be made regarding appropriate resolution cutoffs, whereas the latter can show events or trends during rotation of the crystal (see, for example, Fig. 11 ▶).

### 8.7. Availability

The current version of *autoPROC* is available free of charge to academics, who should go to http://www.globalphasing.com/autoproc/ for further details. Questions about *autoPROC* should be sent to proc-develop@globalphasing.com.
